# Homelessness and COVID-19: Leaving No One Behind

**DOI:** 10.5334/aogh.3186

**Published:** 2021-01-27

**Authors:** Massimo Ralli, Andrea Arcangeli, Lucia Ercoli

**Affiliations:** 1 Department of Sense Organs, Sapienza University of Rome, Italy; 2 Primary Care Services, Eleemosynaria Apostolica, Vatican City State, Italy; 3 Directorate of Health and Hygiene, Vatican City State, Italy; 4 Department of Emergency, Anesthesiology and Resuscitation Sciences, Fondazione Policlinico Universitario A. Gemelli IRCCS, Rome, Italy; 5 Department of Biomedicine and Prevention, Tor Vergata University, Rome, Italy

## Abstract

The United Nations 2030 Agenda for Sustainable Development promotes the “Leaving no one behind” principle and sets goals in areas of critical importance. This principle has become extraordinarily important during the COVID-19 pandemic, and is especially relevant for fragile populations, such as people experiencing homelessness.

Homeless persons live in congregate and poor hygiene settings that may favor virus transmission, often have underling physical and mental comorbidities that place them at high risk of severe forms of COVID-19, and have limited access to public healthcare and social services. In addition, the homeless are often overlooked by safety and health monitoring actions. All of these factors, taken together, place homeless persons at high risk of being left behind.

It is therefore of utmost importance to put in place adequate public health measures to limit spread of infection among homeless persons, rapidly identify and isolate asymptomatic and minimally symptomatic subjects, promptly and appropriately treat positive cases, and correctly handle the entire socioeconomic environment of vulnerable people.

**To the Editor:**

The United Nations 2030 Agenda for Sustainable Development promotes the “Leaving no one behind” principle and sets goals and targets in areas of critical importance for humanity and the planet [1]. This principle has become extraordinarily important during the novel coronavirus disease 19 (COVID-19) pandemic. It is especially relevant for disadvantaged fractions of the population, such as people experiencing homelessness.

Homeless persons present several unique vulnerabilities that have been greatly amplified due to the socioeconomic situation created by the pandemic [2]. The homeless often live in the streets or in high-density congregate settings that may not guarantee basic hygiene and protection practices and adequate social distancing. They have limited access to health care and social services. They are often overlooked by safety and health monitoring actions as they are hard to reach, often transient, and less compliant with prevention measures. All of these factors favor the spread of Severe Acute Respiratory Syndrome Coronavirus 2 (SARS-CoV-2) infection [3]. In addition, people experiencing homelessness often have underling physical and mental comorbidities that place them at high risk of severe forms of COVID-19 and death. Studies have shown that even apart from COVID-19, homeless persons have a mortality up to 10 times higher than the general population, and COVID-19 may be expected to further widen this mortality gap [3]. Finally, lockdown measures adopted at a local or national level to limit contagion add additional difficulties in fulfilling basic human needs of the homeless population, thus further increasing inequalities [3].

All of these factors, taken together, place homeless persons at high risk of being left behind by public health and social services, especially in these weeks in which many countries are facing an increased number of cases in the second wave of COVID-19. This risk may be exacerbated by the fact that a large part of COVID-19 cases are asymptomatic or minimally symptomatic, which leads to delayed diagnosis and late isolation of positive patients, who may then involuntarily spread infection in shelters or among their contacts with dramatic consequences on individual and public health [4].

It is therefore of utmost importance to put in place adequate public health measures that will limit spread of infection among homeless persons, rapidly identify and isolate asymptomatic and minimally symptomatic subjects, promptly and appropriately treat positive cases, and correctly handle the entire socioeconomic environment of vulnerable people. The actions adopted by the Vatican City State to meet these goals in the provision of primary care services to homeless people through the Offices of Papal Charities (Eleemosynaria Apostolica) include:

- deployment of strict hygienic and behavioral regulations to prevent contagion among sheltered and, when possible, unsheltered homeless persons, such as daily symptom check, temperature monitoring, and distribution and use of face masks and hand disinfectants;
- constant education on how infection spreads and on methods to prevent contagion;
- redistribution and reorganization of space in personal and communal shelter areas to guarantee interpersonal distance;
- conducting routine surveillance campaigns among people living in the streets and in homeless shelters using Real-Time Polymerase Chain Reaction (RT-PCR) nasopharyngeal swabs or rapid antigen tests for SARS-CoV-2; and
- advance preparation of facilities to guarantee housing for unsheltered homeless people in the event of temporary closures of homeless shelters during unexpected outbreaks [5].

Governments, national and local, and their social and health departments should collaborate in the deployment of prevention and control services to limit spread of COVD-19 infection in all segments of the population, and most especially among vulnerable groups such as people experiencing homelessness. No one should be left behind.
